# Correlation between biochemical and clinical hyperandrogenism parameter in polycystic ovary syndrome in relation to age

**DOI:** 10.1186/s12902-023-01346-x

**Published:** 2023-04-23

**Authors:** Zaixin Guo, Fengjun Jin, Shuwen Chen, Pan Hu, Yanfang Hao, Qi Yu

**Affiliations:** Department of Obstetrics and Gynecology, Peking Union Medical College Hospital, Peking Union Medical College and Chinese Academy of Medical Sciences, Beijing, China

**Keywords:** Age, Dehydroepiandrosterone, Hyperandrogenism, Polycystic ovary syndrome

## Abstract

**Background:**

To assess the correlation between clinical and biochemical hyperandrogenism parameters in polycystic ovary syndrome (PCOS) according to age.

**Methods:**

This prospective study included 256 PCOS patients diagnosed according to the Rotterdam criteria in a university-based hospital. Androgen levels were measured using liquid chromatography-tandem mass spectrometry. Hirsutism, acne, and alopecia were assessed using the modified Ferriman-Gallwey (mF-G) score, Comprehensive Acne Severity Scale (CASS), and the Ludwig scale, respectively. The correlation between biochemical and clinical hyperandrogenism parameters was assessed in younger and older women with PCOS.

**Results:**

The 256 PCOS patients were classified by age into two groups: age 18–29 years (*n* = 151) and age 30–40 years (*n* = 84). In women with PCOS, mF-G was significantly positively correlated with the free androgen index (FAI), dehydroepiandrosterone (DHEA), and DHEA sulfate (DHEA-S). CASS had a significant positive correlation with DHEA. mF-G was positively correlated with FAI in those aged 18–29 years, but the correlations were not significant in those aged 30–40 years. The positive correlation between specific body regions of clinical hyperandrogenism, especially mF-G of chin, lower abdomen, and thighs, and testosterone, as well as with FAI, was highest in those aged 18–29 years. In those aged 30–40 years clinical hyperandrogenism was mainly affected by DHEA, DHEA-S, and dihydrotestosterone.

**Conclusion:**

The correlation between biochemical and clinical hyperandrogenism parameters varied with age in our East Asian population. Clinical hyperandrogenism was positively correlated with FAI in younger women with PCOS. The correlation between biochemical and clinical hyperandrogenism was not significant in older women with PCOS.

**Supplementary Information:**

The online version contains supplementary material available at 10.1186/s12902-023-01346-x.

## Background

Polycystic ovary syndrome (PCOS) is one of the most common endocrinopathies in women of reproductive age and is characterized by clinical and/or biochemical hyperandrogenism, anovulation, and polycystic ovaries [1, 2]. Among these features, clinical and biochemical hyperandrogenism is considered the cornerstone of PCOS by the Androgen Excess Society [3]. Clinical hyperandrogenism is usually characterized by hirsutism, acne, and alopecia [4]. Biochemical hyperandrogenism is diagnosed based on high levels of serum total testosterone, free testosterone, free androgen index (FAI), androstenedione, dihydrotestosterone (DHT), dehydroepiandrosterone (DHEA), and DHEA sulfate (DHEA-S) [5]. It is important to confirm the associations between the endocrine and clinical characteristics of PCOS, as they influence the follow-up and management of the condition [6]. The relationship between clinical and biochemical hyperandrogenism has been evaluated in many studies, with conflicting results, although biochemical hyperandrogenism modulates the biological mechanism regulating clinical hyperandrogenism [7]. This heterogeneity may be due to age, race, body mass index, insulin sensitivity, variability in PCOS diagnosis, and the method of hormonal assay [8–10].

The presence of androgen excess is highly age-dependent, both in women with and without PCOS [10–12]. This is partly because ovarian steroid secretion capacity starts to decline as early as at age 30 years [13]. Previous studies have shown that total testosterone, free testosterone, and DHEA-S levels are lower in older women with PCOS [12]. In addition, the prevalence of acne and hirsutism showed a significant negative association with age in women with PCOS [12]. Thus, age is an essential factor influencing clinical and biochemical hyperandrogenism. The association between clinical and biochemical hyperandrogenism could vary with age, as age-related changes in clinical and biochemical hyperandrogenism may differ, which was rarely clarified in previous studies. In this study, we attempted to evaluate the correlation between clinical and biochemical hyperandrogenism in women with PCOS in relation to age groups to further demonstrate the dynamic changes of hyperandrogenic characteristics of PCOS.

## Methods

### Study design and participants

This study was approved by the Ethics Committee of Peking Union Medical College Hospital (JS-2852). All women who were diagnosed with PCOS between February 1, 2021, and November 3, 2022, in the gynecological endocrine clinic of Peking Union Medical College Hospital were included in the study. In total, 256 women with PCOS were included in the study.

PCOS diagnosis was based on the revised Rotterdam criteria. None of the women had endocrine or systemic disorders that could affect reproductive physiology, including hyperprolactinemia, Cushing’s syndrome, androgen-secreting tumors, congenital adrenal hyperplasia, and thyroid dysfunction.

### Assessment

The waist-to-hip ratio was defined as waist girth/hip girth. Body mass index was defined as body weight in kilograms divided by height in meters squared (kg/m^2^). Hirsutism was assessed by the modified Ferriman-Gallwey (mF-G) score [14, 15]. Acne was evaluated using the Comprehensive Acne Severity Scale (CASS) [16]. Alopecia was assessed using the Ludwig scale [17]. Baseline blood samples were collected between days 3 and 7 after spontaneous or progestogen-induced bleeding episodes in patients with PCOS. Serum follicle-stimulating hormone, luteinizing hormone, sex hormone binding globulin (SHBG), and prolactin levels were measured using an enzyme immunoassay (Beckman). Serum total testosterone, androstenedione, DHEA, DHEA-S, 17α-hydroxyprogesterone (17-OH PRG), and DHT were measured via liquid–liquid extraction (methyl tertiary-butyl ether) of serum via liquid chromatography-tandem mass spectrometry (LC–MS/MS). FAI was determined as follows: FAI = total testosterone (nmol/L) × 100/SHBG (nmol/L).

### Statistical analysis

We evaluated the correlation between biochemical parameters and the mF-G score, CASS, and Ludwig scale using Pearson’s correlation coefficients with a two-tailed significance test. We used the χ2 and Fisher’s exact tests to compare the categorical variables and analysis of variance to compare the continuous variables. For multiple regression, the best subsets regression (BSR) was used to select best variables, and the final selected combination for BSR was determined by the minimum Bayesian information criterion.

## Results

Supplement Table 1 shows the comparison of clinical characteristics between the 18–29 and 30–40 year-old groups. PCOS patients aged 18–29 years old presented with a significantly higher mF-G score (4.63 vs. 3.12, *P* = 0.002) than PCOS patients aged 30–40 years, especially in the lower abdomen (0.90 vs. 0.57, *P* = 0.007), upper arms (0.36 vs. 0.08, *P* < 0.001), upper back (0.20 vs. 0.04, *P* = 0.013), and lower back (0.29 vs. 0.10, *P* = 0.017). Moreover, PCOS patients aged 18–29 years presented with increased acne, especially in the face and chest. The younger patients had higher levels of biochemical hyperandrogenism. Of these parameters, androstenedione, DHEA, and DHEA-S levels were significantly higher among the younger patients.

Table 1 shows the correlation between mF-G levels and biochemical hyperandrogenism. Considering all 256 study participants, mF-G was positively correlated with FAI (r = 0.316, *P* < 0.001), DHEA (r = 0.145, *P* = 0.02), and DHEA-S (r = 0.142, *P* = 0.023). After selection by BSR, multiple regression showed that mF-G was associated with FAI (beta = 0.20, t = 5.05, *p* < 0.001) and DHEA (beta = 0.16, t = 2.36, *p* = 0.019) in all study participants. In those aged 18–29 years (*n* = 164), mF-G was positively correlated with FAI (r = 0.353, *P* < 0.001). However, mF-G was less positively correlated with DHEA-S (r = 0.206, *P* = 0.048) in those aged 30–40 years (*n* = 92).

**Table 1:** Correlations between mF-G and biochemical parameters of PCOS

	**Total (*n* = 256)**		**Age**			
			**18–29 years old (*n* = 164)**		**30–40 years old (*n* = 92)**	
**Variables**	**Correlation**	***P*-value**	**Correlation**	***P*-value**	**Correlation**	***P*-value**
Testosterone	0.122	0.05	0.128	0.101	-0.03	0.778
FAI	**0.316**	< 0.001^**^	**0.353**	< 0.001^**^	0.102	0.348
DHEA	**0.145**	0.02^*^	0.099	0.209	0.167	0.112
DHEA-S	**0.142**	0.023^*^	0.093	0.235	0.206	0.048^*^
Androstenedione	-0.008	0.899	-0.053	0.501	-0.003	0.976
DHT	-0.019	0.758	-0.131	0.094	0.174	0.098
17-OH PRG	-0.015	0.81	0.035	0.662	-0.158	0.141

*Abbreviations*: *FAI* free androgen index, *DHEA* dehydroepiandrosterone, *DHEA-S* DHEA sulfate, *DHT* dihydrotestosterone, *17-OH PRG* 17α-hydroxyprogesterone

^*^P < 0.05; ^**^P < 0.001

Table 2 shows the correlation between CASS and biochemical hyperandrogenism. CASS was positively correlated with serum DHEA-S (r = 0.145, *P* = 0.021) in all PCOS women recruited. In either younger or older subgroup of PCOS patients, CASS showed no significant association with biochemical hyperandrogenism. Table 3 shows the correlations between alopecia and biochemical hyperandrogenism. No significant correlation was found in the patients of any age or subgroup.

**Table 2:** Correlations between CASS and biochemical parameters of PCOS

	**Total (*n* = 256)**		**Age**			
			**18–29 years old (*n* = 164)**		**30–40 years old (*n* = 92)**	
**Variables**	**Correlation**	***P*-value**	**Correlation**	***P*-value**	**Correlation**	***P*-value**
Testosterone	0.066	0.29	0.06	0.443	0.011	0.914
FAI	0.04	0.538	0.066	0.414	-0.087	0.425
DHEA	0.111	0.076	0.058	0.463	0.139	0.188
DHEA-S	**0.145**	0.021^*^	0.105	0.181	0.158	0.132
Androstenedione	0.086	0.172	0.106	0.176	-0.022	0.839
DHT	0.097	0.122	0.151	0.054	-0.032	0.761
17-OH PRG	0.119	0.06	0.126	0.109	0.117	0.276

*Abbreviations*: *FAI* free androgen index, *DHEA* dehydroepiandrosterone, *DHEA-S* DHEA sulfate, *DHT* dihydrotestosterone, *17-OH PRG* 17α-hydroxyprogesterone

^*^P < 0.05

**Table 3:** Correlations between the Ludwig scale and biochemical parameters of PCOS

	**Total (*n* = 256)**		**Age**			
			**18–29 years old (*n* = 164)**		**30–40 years old (*n* = 92)**	
**Variables**	**Correlation**	***P*-value**	**Correlation**	***P*-value**	**Correlation**	***P*-value**
Testosterone	-0.068	0.278	-0.058	0.459	-0.123	0.244
FAI	0.076	0.24	0.057	0.477	0.114	0.298
DHEA	-0.007	0.916	-0.07	0.374	0.108	0.307
DHEA-S	0.03	0.632	0.029	0.712	0.018	0.868
Androstenedione	0.029	0.647	0.04	0.613	-0.004	0.971
DHT	0.026	0.677	-0.013	0.865	0.073	0.488
17-OH PRG	-0.044	0.49	0.051	0.515	-0.186	0.082

*Abbreviations*: *FAI* free androgen index, *DHEA* dehydroepiandrosterone, *DHEA-S* DHEA sulfate, *DHT* dihydrotestosterone, *17-OH PRG* 17α-hydroxyprogesterone

**Fig. 1:**
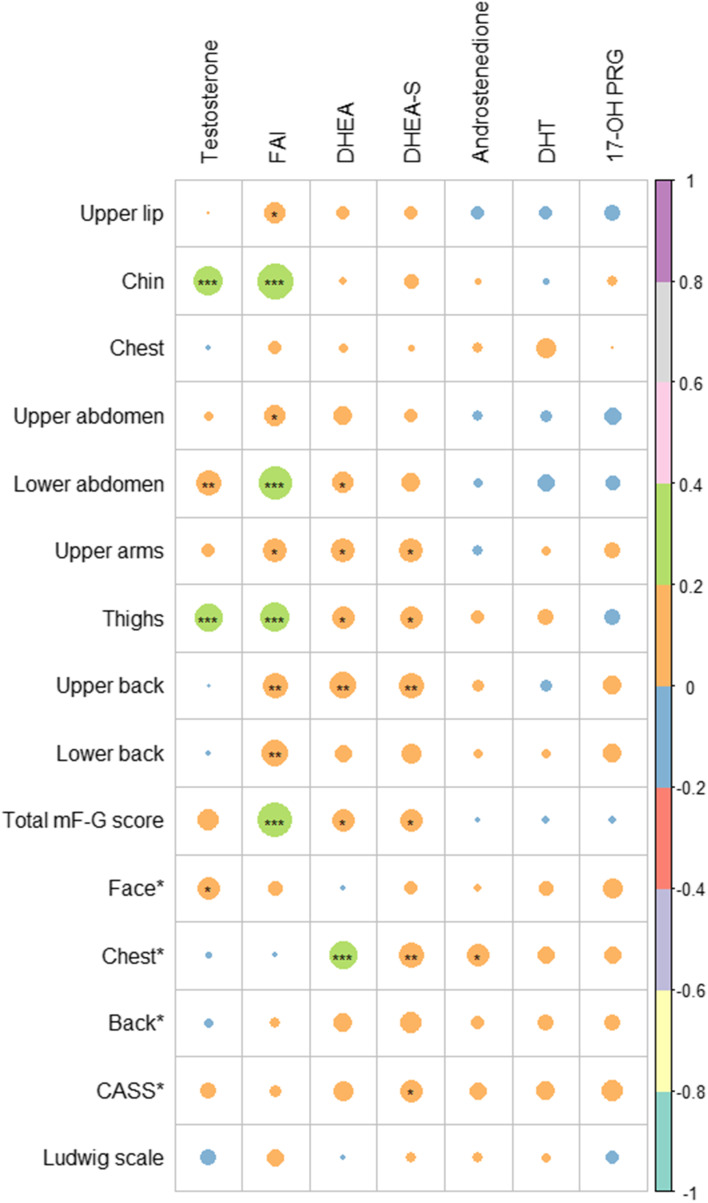
Heatmap showing the correlations between the clinical and biochemical parameters of all patients with PCOS. * Body regions score in CASS. Circle size represents the absolute values of correlation coefficients. Green represents positive correlation with correlation coefficients of 0.2–0.4. Orange represents positive correlation with correlation coefficients of 0–0.2. Blue represents negative correlation with correlation coefficients of 0–0.2. Abbreviations: CASS, Comprehensive Acne Severity Scale; FAI, free androgen index; DHEA, dehydroepiandrosterone; DHEA-S, DHEA sulfate; DHT, dihydrotestosterone; PCOS, polycystic ovary syndrome; 17-OH PRG, 17α-hydroxyprogesterone

Figure 1 shows the correlations between region-specific clinical and biochemical hyperandrogenism. Hirsutism was mainly affected by FAI, as mF-G scores in the chin (r = 0.345, *P *< 0.001), upper abdomen (r = 0.128, *P* = 0.046), lower abdomen (r = 0.294, *P* < 0.001), upper arms (r = 0.151, *P* = 0.018), thighs (r = 0.227, *P* < 0.001), upper back (r = 0.178, *P* = 0.005), and lower back (r = 0.186, *P* = 0.004) were significantly correlated with FAI. Additionally, testosterone, DHEA, and DHEA-S significantly affected hirsutism. FAI and testosterone did not seem to be correlated with CASS, but DHEA, DHEA-S, and androstenedione affected total CASS and chest CASS.

**Fig. 2:**
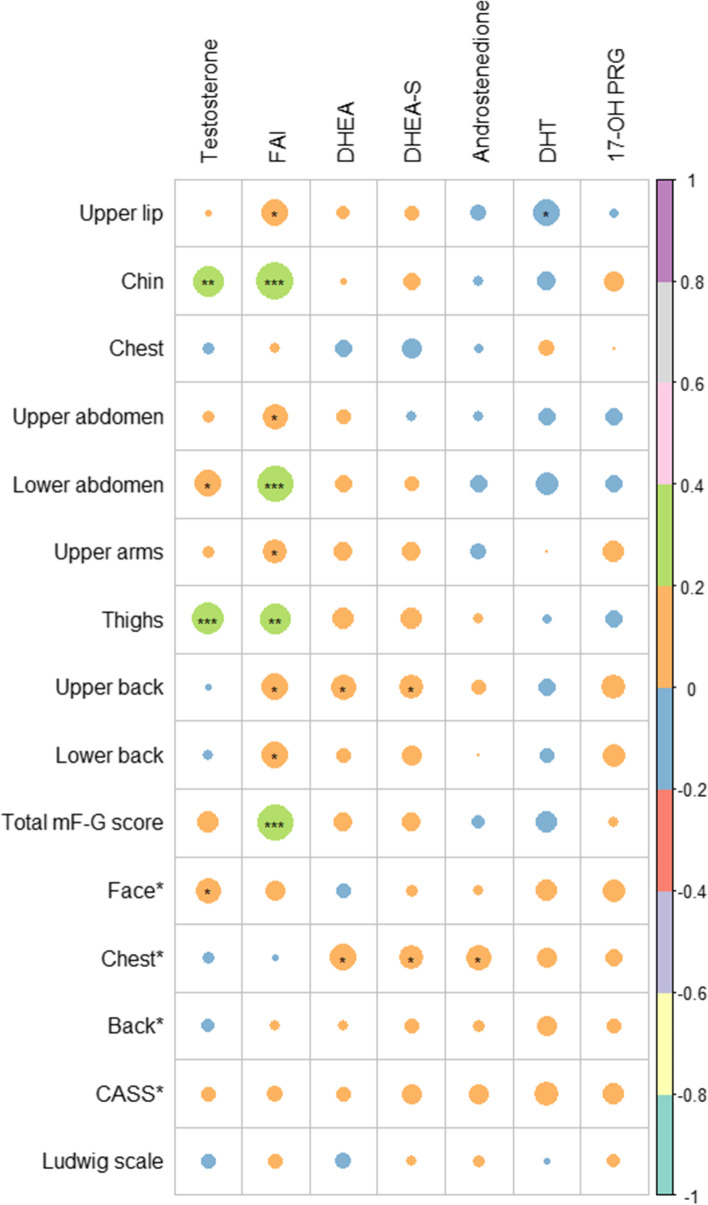
Heatmap showing the correlations between the clinical and biochemical parameters of those with PCOS aged 18–29 years. * Body regions score in CASS. Circle size represents the absolute values of correlation coefficients. Green represents positive correlation with correlation coefficients of 0.2–0.4. Orange represents positive correlation with correlation coefficients of 0–0.2. Blue represents negative correlation with correlation coefficients of 0–0.2. Abbreviations: CASS, Comprehensive Acne Severity Scale; FAI, free androgen index; DHEA, dehydroepiandrosterone; DHEA-S, DHEA sulfate; DHT, dihydrotestosterone; PCOS, polycystic ovary syndrome; 17-OH PRG, 17α-hydroxyprogesterone

**Fig. 3:**
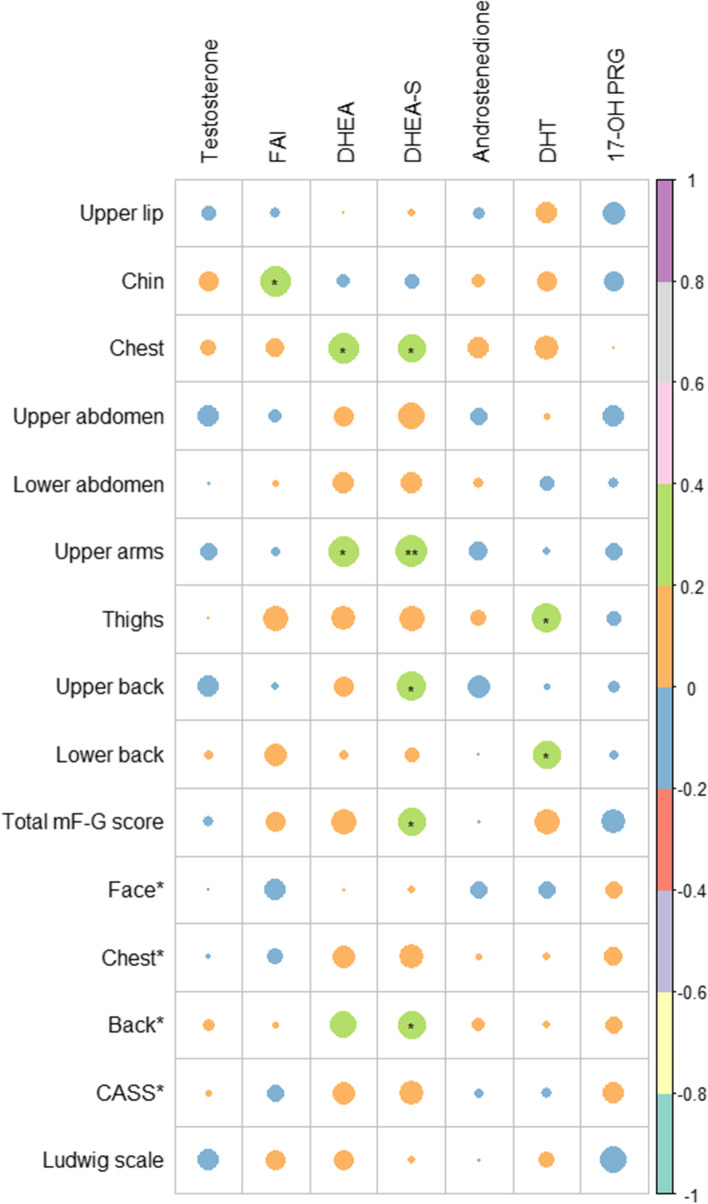
Heatmap showing the correlations between the clinical and biochemical parameters of those with PCOS aged 30–40 years. * Body regions score in CASS. Circle size represents the absolute values of correlation coefficients. Green represents positive correlation with correlation coefficients of 0.2–0.4. Orange represents positive correlation with correlation coefficients of 0–0.2. Blue represents negative correlation with correlation coefficients of 0–0.2. Abbreviations: CASS, Comprehensive Acne Severity Scale; FAI, free androgen index; DHEA, dehydroepiandrosterone; DHEA-S, DHEA sulfate; DHT, dihydrotestosterone; PCOS, polycystic ovary syndrome; 17-OH PRG, 17α-hydroxyprogesterone

Figures 2 and 3 show the correlations between specific regions of clinical hyperandrogenism and biochemical hyperandrogenism in those aged 18–29 and 30–40 years, respectively. In the younger patients, hirsutism was mainly affected by FAI and testosterone levels. For specific body regions, mF-G scores of the chin, lower abdomen, and thighs were correlated with FAI and testosterone, and scores of the upper lip, upper arms, upper back, and lower back were correlated with FAI, with lower r values. In contrast, hirsutism was mainly affected by DHEA, DHEA-S, and DHT in the older patients. For specific body regions, the mF-G score of the chin was mainly affected by FAI; the mF-G scores of the chest, upper arms, and upper back were mainly affected by DHEA and DHEA-S; and the mF-G scores of the thighs and lower back were mainly affected by DHT. For acne, chest CASS was positively correlated with DHEA and DHEA-S in the younger patients, whereas back CASS was positively correlated with DHEA-S in the older patients.

## Discussion

In this study, we investigated the correlation between biochemical and clinical hyperandrogenism parameters in patients with PCOS in East Asia. Specifically, we divided PCOS patients into younger and older groups and found different correlations in the different age groups.

Clinical and biochemical hyperandrogenism are the major characteristics of PCOS. The most common clinical signs of hyperandrogenism are hirsutism, acne, and alopecia. Hirsutism is stimulated by the local elevation of DHT in pilosebaceous units, which is converted from testosterone by 5α-reductase [18]. 5α-reductase is locally affected by androgens, insulin, and insulin-like growth factors [19]. Acne production is stimulated by androgens, which bind to androgen receptors on pilosebaceous units, and result in sebaceous gland hyperplasia, increased sebum production, and abnormal follicular epithelial cell keratinization [20–22]. Alopecia results from excess androgen, as androgens decrease the percentage of anagen hairs by converting terminal follicles to vellus hair, which is infrequent in women [23, 24]. Thus, clinical hyperandrogenism is influenced by circulating androgen levels, together with local androgen levels and the sensitivity of hair follicles to androgens, which can differ by age, race, ethnicity, and familial background [12, 25].

The relationship between clinical and biochemical hyperandrogenism has been investigated in many studies, with conflicting results. A meta-analysis, which included 35 studies and 6593 PCOS patients, showed significant positive relationships between mF-G score and androstenedione and DHEA-S [7]. However, this finding was limited by variabilities in PCOS diagnosis, race, ethnicity, and interpretation of hirsutism among the included patients. In addition, test methods could also have impacted the results, as total testosterone measured by LC–MS/MS and not by chemiluminescent immunoassay was found to be a strong predictor of hirsutism in an East Asian population [26]. Our results showed that mF-G was correlated with FAI, DHEA, and DHEAS. Among these biochemical markers, FAI, which reflects free testosterone, was the most powerful predictor of hirsutism, with a correlation coefficient of 0.316. Regarding specific body areas, FAI was well correlated with the chin, lower abdomen, and thigh. Acne and alopecia are not usually evaluated for the association between clinical and biochemical hyperandrogenism because methods to precisely assess these parameters are lacking [27]. This study used CASS to evaluate acne and found that acne was correlated with DHEA-S.

Age profoundly affects the relationship between clinical and biochemical androgen characteristics, because of an age-related decrease in androgen secretion. The presence of excess androgen was significantly different between PCOS patients aged < 30 and ≥ 30 years, which was proven both in the current study and in a previous study [12]. Thus, clinical and biochemical relationships also differ between PCOS patients aged < 30 and ≥ 30 years. In our study, the mF-G score was correlated with FAI and DHEA in patients aged 18–29 years. However, the mF-G score showed no correlation with biochemical androgen levels in patients aged 30–40 years. Concerning specific body areas, hirsutism, excluding the chest, was affected by FAI in patients aged 18–29 years, especially in the chin, lower abdomen, and thighs. In contrast, in patients aged 30–40 years, hirsutism in the chest, upper arm, and upper back was mainly affected by DHEA and DHEAS, while hirsutism in the thigh was mainly affected by DHT. It is noteworthy that FAI had a lesser effect on hirsutism with increasing age. This probably resulted from the decline in ovarian steroid secretion from the age of 30 years, as the ovary is an important source of testosterone [10]. Ovarian steroid secretory capacity is determined by age-related follicular viability, which is influenced by genetic factors, cellular metabolism, and environmental factors [28–30]. As ovarian primordial and small antral follicles decrease with age, inhibin B decreases, resulting in increased FSH, which is hypothesized as a stimulus to maintain circulating estradiol, and testosterone as a substrate for estrogen biosynthesis is used [10, 13, 31]. Also, the levels of androstenedione, DHEA, and DHEAS, which are mainly produced by the adrenal gland, decrease with aging due to a decrease in 17,20-lyase activity [32].

The present study had several strengths. First, the clinical hyperandrogenic features in this prospective study were recorded by a single doctor, thus decreasing inter-observer bias. Second, we evaluated our biochemical androgens using LC–MS/MS methods, which are less affected by structurally related steroid metabolites [33]. Third, we evaluated the relationship between clinical and biochemical hyperandrogenism features in different age ranges and demonstrated the profound impact of age on the relationship. Fourth, we focused on specific body regions for clinical features, which ensures thorough and concrete evaluation.

This study has some limitations. First, our study was performed at a single center with a limited population. Second, the results only included the East Asian population and may not apply to other ethnicities. Third, free testosterone levels were not measured in our patients. Free testosterone is an important and accurate index for evaluating biochemical hyperandrogenism as recommended by the international evidence-based guideline for the assessment and management of PCOS 2018 [34]. Measurement of free testosterone would have ensured a more robust evaluation.

## Conclusions

The correlation between biochemical and clinical hyperandrogenism parameters depended on age. The correlation between biochemical and clinical hyperandrogenism was more significant in younger patients with PCOS. FAI played a fundamental role in clinical hyperandrogenism in younger women with PCOS, whereas DHEA, DHEA-S, and DHT played more important roles in older women with PCOS.

## Supplementary Information

**Additional file 1: Supplement Table 1.** Comparison of the clinical characteristics of the women with PCOS in the 18–29 and 30–40 year-old groups.

## Data Availability

The datasets used in the current study are available from the corresponding author on reasonable request.

## Abbreviations

BSR: Best subsets regression
CASS: Comprehensive Acne Severity Scale
DHEA: Dehydroepiandrosterone
DHEA-S: Ehydroepiandrosterone sulfate
FAI: Free androgen index
LC–MS/MS: Liquid chromatography-tandem mass spectrometry
mF-G: Modified Ferriman-Gallwey
PCOS: Polycystic ovary syndrome
SHBG: Sex hormone binding globulin

## References

[1] Yildiz BO, Bozdag G, Yapici Z, Esinler I, Yarali H (2012). Prevalence, phenotype and cardiometabolic risk of polycystic ovary syndrome under different diagnostic criteria. Hum Reprod.

[2] Escobar-Morreale HF (2018). Polycystic ovary syndrome: definition, aetiology, diagnosis and treatment. Nat Rev Endocrinol.

[3] Azziz R, Carmina E, Dewailly D, Diamanti-Kandarakis E, Escobar-Morreale HF, Futterweit W, Janssen OE, Legro RS, Norman RJ, Taylor AE (2006). Positions statement: criteria for defining polycystic ovary syndrome as a predominantly hyperandrogenic syndrome: an Androgen Excess Society guideline. J Clin Endocrinol Metab.

[4] Panidis D, Tziomalos K, Papadakis E, Chatzis P, Kandaraki EA, Tsourdi EA, Vosnakis C, Katsikis I (2013). The clinical significance and primary determinants of hirsutism in patients with polycystic ovary syndrome. Eur J Endocrinol.

[5] Coskun A, Ercan O, Arikan DC, Özer A, Kilinc M, Kiran G, Kostu B (2011). Modified Ferriman-Gallwey hirsutism score and androgen levels in Turkish women. Eur J Obstet Gynecol Reprod Biol.

[6] Cibula D, Hill M, Starka L (2000). The best correlation of the new index of hyperandrogenism with the grade of increased body hair. Eur J Endocrinol.

[7] Amiri M, Ramezani Tehrani F, Nahidi F, Bidhendi Yarandi R, Behboudi-Gandevani S, Azizi F (2017). Association between biochemical hyperandrogenism parameters and Ferriman-Gallwey score in patients with polycystic ovary syndrome: A systematic review and meta-regression analysis. Clin Endocrinol (Oxf).

[8] ESHRE/ASRM: Consensus on infertility treatment related to polycystic ovary syndrome. Hum Reprod (Oxford, England) 2008, 23(3):462–477. 10.1093/humrep/dem426 18308833

[9] Amato MC, Galluzzo A, Merlino S, Mattina A, Richiusa P, Criscimanna A, Giordano C (2006). Lower insulin sensitivity differentiates hirsute from non-hirsute Sicilian women with polycystic ovary syndrome. Eur J Endocrinol.

[10] Piltonen T, Koivunen R, Perheentupa A, Morin-Papunen L, Ruokonen A, Tapanainen JS (2004). Ovarian age-related responsiveness to human chorionic gonadotropin in women with polycystic ovary syndrome. J Clin Endocrinol Metab.

[11] Winters SJ, Talbott E, Guzick DS, Zborowski J, McHugh KP (2000). Serum testosterone levels decrease in middle age in women with the polycystic ovary syndrome. Fertil Steril.

[12] Liang SJ, Hsu CS, Tzeng CR, Chen CH, Hsu MI (2011). Clinical and biochemical presentation of polycystic ovary syndrome in women between the ages of 20 and 40. Hum Reprod.

[13] Piltonen T, Koivunen R, Ruokonen A, Tapanainen JS (2003). Ovarian age-related responsiveness to human chorionic gonadotropin. J Clin Endocrinol Metab.

[14] Ferriman D, Gallwey JD (1961). Clinical assessment of body hair growth in women. J Clin Endocrinol Metab.

[15] Hatch R, Rosenfield RL, Kim MH, Tredway D (1981). Hirsutism: implications, etiology, and management. Am J Obstet Gynecol.

[16] Tan JK, Tang J, Fung K, Gupta AK, Thomas DR, Sapra S, Lynde C, Poulin Y, Gulliver W, Sebaldt RJ (2007). Development and validation of a comprehensive acne severity scale. J Cutan Med Surg.

[17] Ludwig E (1977). Classification of the types of androgenetic alopecia (common baldness) occurring in the female sex. Br J Dermatol.

[18] Archer JS, Chang RJ (2004). Hirsutism and acne in polycystic ovary syndrome. Best Pract Res Clin Obstet Gynaecol.

[19] Falsetti L, Gambera A, Andrico S, Sartori E (2002). Acne and hirsutism in polycystic ovary syndrome: clinical, endocrine-metabolic and ultrasonographic differences. Gynecol Endocrinol.

[20] Borgia F, Cannavò S, Guarneri F, Cannavò SP, Vaccaro M, Guarneri B (2004). Correlation between endocrinological parameters and acne severity in adult women. Acta Derm Venereol.

[21] Timpatanapong P, Rojanasakul A (1997). Hormonal profiles and prevalence of polycystic ovary syndrome in women with acne. J Dermatol.

[22] Thiboutot D, Chen W (2003). Update and future of hormonal therapy in acne. Dermatology.

[23] Yildiz BO (2006). Diagnosis of hyperandrogenism: clinical criteria. Best Pract Res Clin Endocrinol Metab.

[24] Carmina E, Rosato F, Jannì A, Rizzo M, Longo RA (2006). Extensive clinical experience: relative prevalence of different androgen excess disorders in 950 women referred because of clinical hyperandrogenism. J Clin Endocrinol Metab.

[25] Carmina E, Koyama T, Chang L, Stanczyk FZ, Lobo RA (1992). Does ethnicity influence the prevalence of adrenal hyperandrogenism and insulin resistance in polycystic ovary syndrome?. Am J Obstet Gynecol.

[26] Yang Y, Ouyang N, Ye Y, Hu Q, Du T, Di N, Xu W, Azziz R, Yang D, Zhao X (2020). The predictive value of total testosterone alone for clinical hyperandrogenism in polycystic ovary syndrome. Reprod Biomed Online.

[27] Escobar-Morreale HF, Carmina E, Dewailly D, Gambineri A, Kelestimur F, Moghetti P, Pugeat M, Qiao J, Wijeyaratne CN, Witchel SF (2012). Epidemiology, diagnosis and management of hirsutism: a consensus statement by the Androgen Excess and Polycystic Ovary Syndrome Society. Hum Reprod Update.

[28] Faddy M (2000). Follicle dynamics during ovarian ageing. Mol Cell Endocrinol.

[29] Westhoff C, Murphy P, Heller D (2000). Predictors of ovarian follicle number. Fertil Steril.

[30] Jones K (2008). Meiosis in oocytes: predisposition to aneuploidy and its increased incidence with age. Hum Reprod Update.

[31] Burger HG, Hale GE, Dennerstein L, Robertson DM (2008). Cycle and hormone changes during perimenopause: the key role of ovarian function. Menopause.

[32] Laughlin G, Barrett-Connor E (2000). Sexual dimorphism in the influence of advanced aging on adrenal hormone levels: the Rancho Bernardo Study. J Clin Endocrinol Metab.

[33] Handelsman DJ, Teede HJ, Desai R, Norman RJ, Moran LJ (2017). Performance of mass spectrometry steroid profiling for diagnosis of polycystic ovary syndrome. Hum Reprod.

[34] Teede H, Misso M, Costello M, Dokras A, Laven J, Moran L, Piltonen T, Norman R (2018). Recommendations from the international evidence-based guideline for the assessment and management of polycystic ovary syndrome. Human reproduction (Oxford, England).

