# Supplementary material for: Correlation between biochemical and clinical hyperandrogenism parameter in polycystic ovary syndrome in relation to age
Source: BMC Endocr Disord. 2023 Apr 23;23:89. doi: 10.1186/s12902-023-01346-x (PMC10122797; doi:10.1186/s12902-023-01346-x)
Supplement: Supplementary file 1 — Additional file 1: Supplement Table 1. Comparison of the clinical characteristics of the women with PCOS in the 18–29 and 30–40 year-old groups. [file 12902_2023_1346_MOESM1_ESM.docx]

**Supplement Table 1** Comparison of the clinical characteristics of the women with PCOS in the 18–29 and 30–40 year-old groups.

|  | **Total**  **(n=256)** | **Age** | | **P-value^#^** |
| --- | --- | --- | --- | --- |
|  |  | **18–29 years old (n=164)** | **30-40 years old (n=92)** |  |
| Age (years) | 27.34±5.08 | 24.33±3.31 | 32.71±2.72 | **<0.001***** |
| Body mass index (kg/m^2^) | 24.10±4.57 | 23.53±4.30 | 25.12±4.88 | **0.007**** |
| Waist-hip ratio (mean±SD) | 0.81±0.06 | 0.81±0.06 | 0.83±0.06 | **0.03*** |
| Menarche (years) | 12.94±1.32 | 12.91±1.33 | 12.99±1.30 | 0.663 |
| Number of menstrual cycles per year (n) | 7.21±3.68 | 7.23±3.87 | 7.44±3.39 | 0.68 |
| **Endocrine** | | | | |
| AMH (ng/ml) | 9.77±6.58 | 10.33±6.30 | 8.76±6.98 | 0.073 |
| Baseline FSH (IU/L) | 6.38±1.86 | 6.41±1.84 | 6.34±1.91 | 0.776 |
| Baseline LH (IU/L) | 12.49±9.10 | 13.36±10.01 | 10.96±6.98 | 0.053 |
| Baseline LH/FSH | 2.01±1.36 | 2.15±1.51 | 1.77±1.03 | **0.046*** |
| Baseline PRL (ng/mL) | 13.65±7.41 | 14.15±7.54 | 12.77±7.11 | 0.176 |
| **Clinical Hyperandrogenism** | | | | |
| **mF-G score** |  |  |  |  |
| Upper lip | 1.54±1.13 | 1.59±1.13 | 1.43±1.11 | 0.287 |
| Chin | 0.32±0.75 | 0.39±0.80 | 0.21±0.62 | 0.059 |
| Chest | 0.35±0.53 | 0.35±0.53 | 0.35±0.52 | 0.932 |
| Upper abdomen | 0.31±0.67 | 0.37±0.73 | 0.21±0.52 | 0.056 |
| Lower abdomen | 0.78±0.94 | 0.90±1.05 | 0.57±0.67 | **0.007**** |
| Upper arms | 0.26±0.58 | 0.36±0.67 | 0.08±0.27 | **<0.001***** |
| Thighs | 0.17±0.45 | 0.18±0.49 | 0.14±0.38 | 0.479 |
| Upper back | 0.14±0.47 | 0.20±0.56 | 0.04±0.21 | **0.013*** |
| Lower back | 0.22±0.61 | 0.29±0.71 | 0.10±0.33 | **0.017*** |
| Total mF-G score | 4.09±3.76 | 4.63±4.26 | 3.12±2.38 | **0.002**** |
| **CASS** |  |  |  |  |
| Face | 1.38±1.09 | 1.51±1.10 | 1.14±1.03 | **0.01*** |
| Chest | 0.57±0.79 | 0.66±0.83 | 0.40±0.70 | **0.011*** |
| Back | 0.39±0.68 | 0.41±0.69 | 0.36±0.67 | 0.576 |
| Total CASS score | 2.34±1.95 | 2.58±2.01 | 1.90±1.76 | **0.007**** |
| **Ludwig scale** | 0.56±0.60 | 0.58±0.61 | 0.53±0.60 | 0.554 |
| **Biochemical Hyperandrogenism** | | | | |
| Testosterone (ng/dl) | 0.43±0.30 | 0.45±0.34 | 0.38±0.20 | 0.072 |
| FAI | 5.63±5.64 | 5.98±6.25 | 5.01±4.26 | 0.203 |
| Androstenedione (ng/dl) | 1.93±0.76 | 2.02±0.73 | 1.78±0.79 | **0.014*** |
| DHEA (ng/dl) | 5.70±3.11 | 6.16±3.25 | 4.88±2.67 | **0.001***** |
| DHEA-S (ng/dl) | 2065.39±906.94 | 2167.38±879.76 | 1883.58±930.81 | **0.016*** |
| 17-OH PRG (ng/dl) | 0.77±0.56 | 0.76±0.51 | 0.77±0.64 | 0.984 |
| DHT (ng/dl) | 0.08 (0.06) | 0.09 (0.05) | 0.07 (0.06) | 0.071 |

*Abbreviations*: AMH, anti-müllerian hormone; FSH, follicle stimulating hormone; LH, luteinizing hormone; PRL, prolactin; HOMA-IR, homeostasis model assessment of insulin resistance; LDL-C, low density lipoprotein cholesterol; HDL-C, high-density lipoprotein cholesterol; SHBG, sex hormone-binding globulin; FAI, free androgen index; DHEA, dehydroepiandrosterone; DHEA-S, DHEA sulfate; DHT, dihydrotestosterone; 17-OH PRG, 17α-hydroxyprogesterone.

*P<0.05; **P<0.01; ***P<0.001

^#^ PCOS patients of age 18–29 years old versus 30-40 years old
